# Biological Maturation and Physical Performance in Youth Football: Associations Across Professional and Non-Professional Environments

**DOI:** 10.3390/jfmk11030257

**Published:** 2026-06-29

**Authors:** Manuel Amore, Maria Francesca Piacentini, Vincenzo Sorgente, Francesco Sera, Diego Minciacchi

**Affiliations:** 1 Department of Movement, Human and Health Sciences, University of Rome ‘Foro Italico’, 00135 Rome, Italy; mariafrancesca.piacentini@uniroma4.it; 2 Kinesiology and Motor Control (Ki.Mo.Co.) Laboratory, Department of Experimental and Clinical Medicine, Physiological Sciences Section, University of Florence, 50134 Florence, Italy; vincenzo.sorgente@unifi.it; 3 Department of Statistics, Computer Science and Applications G. Parenti, University of Florence, 50121 Florence, Italy; francesco.sera@unifi.it

**Keywords:** biological maturation, youth football, physical performance, training environment, talent identification, long-term athlete development

## Abstract

**Background**: Biological maturation is a major determinant of physical performance in youth football, although the previous evidence suggests that training context may influence maturation–performance associations. This study investigated the association between biological maturation and physical performance in youth football players from professional and non-professional settings. **Methods**: A total of 302 male football players (Under-10 to Under-14) from a professional academy (*n* = 122) and non-professional clubs (*n* = 180) participated. Biological maturation was estimated using maturity offset and age at peak height velocity (aPHV). Physical performance was assessed through standing broad jump, T-test agility, and sit-and-reach tests. General Linear Models and stratified correlation analyses were used to examine the interaction between maturation, age category, and training environment. Relative age distribution was also described. **Results**: Professional academy players demonstrated superior explosive power, agility, and flexibility across most age categories compared with non-professional players. Significant associations between biological maturation and physical performance were observed mainly in the non-professional environment, particularly for agility and explosive power, whereas few significant relationships emerged in the professional academy. Significant interactions between training environment, age category, and maturation status were found for all performance measures, with the strongest effect observed for agility. A relative age effect emerged only in the older professional categories. **Conclusions**: Associations between biological maturation and physical performance differed according to training environment in youth football players. Stronger maturation–performance relationships were generally observed in non-professional settings, whereas weaker associations emerged in the professional academy. However, due to the cross-sectional design and the likely interdependence between maturation, selection, and training exposure, causal interpretations cannot be inferred.

## 1. Introduction

The development of youth football players is a multifactorial process shaped by the complex interaction of biological maturation, physical performance, psychosocial factors, and the training environment, all of which contribute to talent identification and long-term athletic development [1]. Among these factors, biological maturation—defined as the process of progressing towards the adult state, characterized by its timing (when specific maturational events occur) and tempo (the rate at which those events progress) relative to chronological age—has been consistently identified as a major determinant of short-term physical performance and selection outcomes (e.g., recruitment into professional academies, squad selection for competitive matches, and inclusion in talent identification programs) in youth football [2,3,4]. Indicators such as age at peak height velocity (aPHV) are commonly used to estimate maturation status and have been shown to influence anthropometric characteristics, neuromuscular development, and physical performance during childhood and adolescence [5,6,7]. The individual variability in these maturational processes often interacts with the organizational structure of youth sports, giving rise to systemic selection biases.

A substantial body of research has documented the presence of maturity-associated variation and the relative age effect (RAE)—defined as the chronological age difference between children born within the same selection year—in youth football, whereby early maturing players are overrepresented in talent identification and selection processes [7,8,9,10]. Early maturers typically display temporary physical advantages, including greater stature, strength, speed, and power, which may be misinterpreted as indicators of superior football potential [3,11,12]. This phenomenon, often described as maturity bias, can lead to the systematic exclusion of later-maturing players, despite evidence suggesting that late maturers may ultimately reach comparable or even superior performance levels in adulthood [6,8,13,14]. Such findings challenge the validity of talent identification models that prioritize short-term physical performance over long-term developmental potential [15,16].

In youth football, early maturing players face heightened pressure and burnout risks, whereas later-maturing peers often develop greater resilience and adaptive coping strategies [17,18]. Ultimately, the extent to which these biological and psychosocial factors translate into performance outcomes depends heavily on the training environment [19].

Professional youth academies are typically characterized by structured training programs, systematic physical preparation, and early performance-based selection. This high-level training and the pre-selection of physically elite individuals may lead to a homogenization of the group, which could attenuate or mask the influence of biological maturation on performance outcomes compared to less selective environments [20]. In contrast, non-professional or amateur environments often present greater heterogeneity in training exposure and physical preparation, potentially allowing maturation-related differences to exert a stronger influence on observable performance [21]. However, despite increasing recognition of the role of contextual factors, comparative analyses examining how biological maturation interacts with different training environments remain limited, particularly in youth football populations spanning pre- and circa-aPHV stages.

This gap is particularly relevant in football systems characterized by early selection and strong institutional structures, where training environment and competitive pressure may substantially influence developmental trajectories [22].

This study posits that the training environment acts as a critical moderator. We hypothesized that maturation–performance associations would differ according to training environment. Clarifying this relationship is essential to determine if elite performance is a product of inherent maturational advantages or high-quality training interventions.

Therefore, the primary purpose of the present study was to examine whether associations between biological maturation and physical performance differed between professional and non-professional youth football environments. Specifically, this study aimed to examine differences in physical performance across age categories, while exploring the interplay between age at peak height velocity (aPHV) and physical performance measures to determine whether this association is influenced by the training context.

As a secondary descriptive objective, we characterized the sample in terms of relative age distribution (RAE) to provide a comprehensive overview of the selection dynamics within the two investigated environments. Ultimately, understanding the moderating role of the training environment provides the necessary tools to distinguish maturational selection bias from true athletic potential, promoting more equitable and effective long-term development pathways.

## 2. Materials and Methods

### 2.1. Participants

A cross-sectional observational design was employed to investigate the interplay between maturation status and athletic performance in youth soccer players. A total of 302 male aged 9–14 years were recruited and stratified into two cohorts: 122 players from a professional youth academy—defined as clubs competing in the top two national divisions (Serie A or Serie B)—and 180 players from non-professional (amateur) clubs, spanning the Under-10 to Under-14 age categories. The anthropometric characteristics stratified by age category and training environment are presented in Table 1. Inclusion criteria required participants to be registered with their respective clubs for at least one full season, free from acute injuries, and without diagnosed growth disorders.

### 2.2. Training Volume and Weekly Schedule

Weekly training schedules were broadly comparable between the professional and non-professional environments with respect to session frequency and nominal duration. For both groups, players in the Under-10 to Under-13 categories participated in three field-based training sessions per week plus one competitive match during the weekend. In the Under-14 category, the training frequency was increased to four sessions per week in addition to the weekly match for both environments. Each training session lasted approximately 90 min. However, it must be acknowledged that detailed quantification of internal load, external load, training intensity, resistance training exposure, coaching quality, and accumulated training history was not available, limiting a more comprehensive comparison of the actual training stimuli between the two environments.

### 2.3. Procedures

Subjects underwent a set of non-invasive measurements of their weight (SECA mechanical column scale MOD. 756, Hamburg, Germany), their height and their sitting height (SECA 206 Wall-Mounted Stadiometer, Hamburg, Germany) to estimate the maturation status and the estimate of aPHV. Leg length was calculated as the difference between stature and sitting height. The aPHV was calculated using Mirwald and colleagues’ equation [23].

Body Mass Index (BMI) was included as a descriptive characteristic to provide a standardized measure of the players’ physical profile relative to their stature and mass. This is particularly relevant in youth football, as body composition and physical robustness significantly influence power-dependent performance tasks and can vary substantially across different maturational stages. However, it does not directly reflect body composition during maturation.

Physical performance tests were selected to assess football-specific attributes. The tests were conducted on the same synthetic surface during the competitive season, at the same time of day, and while wearing soccer footwear. Participants were familiarized with testing procedures prior to data collection. Before physical testing, all participants performed a standardized dynamic warm-up lasting 15 min, consisting of 5 min of low-intensity jogging, followed by dynamic stretching (e.g., leg swings, lunges) and progressive mobilization exercises (e.g., high knees, butt kicks) to minimize injury risk.

#### 2.3.1. Standing Broad Jump Test

Lower-limb explosive power was assessed using the Standing Broad Jump (SBJ) test. Participants were instructed to stand behind a marked take-off line with feet shoulder-width apart. The test required a maximal horizontal jump initiated from a static upright position; however, a preparatory countermovement involving knee flexion and a vigorous bilateral arm swing was permitted to optimize performance. To ensure data validity, participants were required to maintain a stable, upright landing without losing balance or touching the floor with their hands. Distance was measured in centimetres (cm) from the take-off line to the point of contact of the heel closest to the starting line. Each athlete performed three trials, separated by a 1 min rest period to mitigate fatigue, with the furthest successful attempt retained for subsequent statistical analysis [24].

#### 2.3.2. T-Test Agility

Agility and multidirectional speed were assessed using a standardized T-test protocol. The test was conducted on a T-shaped course consisting of four cones: a starting cone (A), a central cone (B) placed 10 m ahead, and two lateral cones (C and D) positioned 5 m to the left and right of the center, respectively. Participants began in a standing position behind the start line at cone A. Upon a self-initiated start, athletes performed a maximal sprint forward to cone B, followed by a lateral shuffle to the left to touch cone C, a lateral shuffle across the course to touch cone D, and a final shuffle back to the center at cone B. The trial concluded with a 10 m backpedal to the starting line. To ensure consistency and technical validity, participants were required to maintain a forward-facing orientation throughout the lateral and reverse phases without crossing their feet. Performance time was recorded to the nearest 0.01 s using a dual-beam photoelectric timing gate system (Witty System, Microgate, Bolzano, Italy). Each athlete completed three maximal trials with a 2 min rest interval between attempts to ensure full recovery, with the fastest time retained for analysis [25].

#### 2.3.3. Sit-and-Reach Test

Hamstring and lower-back flexibility was evaluated using the standardized Sit-and-Reach test. The sit-and-reach test was included to evaluate hamstring and lower back flexibility, which are fundamental components of functional movement screening in youth football even though it should not be interpreted as a direct predictor of football-specific performance. In developing athletes, monitoring flexibility is critical to identify potential muscle-tendon imbalances that often occur during rapid growth phases (i.e., the adolescent growth spurt). Maintaining adequate range of motion is considered essential for injury prevention and for ensuring the correct execution of sport-specific technical actions, rather than as a performance predictor for selection purposes. Participants performed the test in a seated position on the floor with legs fully extended and the soles of their feet placed flat against a professional diagnostic box (Baseline^®^, White Plains, NY, USA). To ensure procedural standardization, the knees were required to be held in full extension throughout the duration of the effort. From the starting position, athletes were instructed to reach forward slowly and maximally along the measuring scale with both hands overlapping, palms facing downward. Participants were required to maintain the reached position for at least two seconds to allow for an accurate recording of the distance. To optimize performance and ensure safety, athletes were encouraged to exhale during the forward reach phase, avoiding any jerky or ballistic movements (i.e., ‘bouncing’). The ‘zero’ point was set at the level of the feet; scores reaching beyond the toes were recorded as positive values, while distances short of the toes were recorded as negative values. Each participant performed three trials, separated by a 30 s recovery period, with the highest score (cm) retained for final analysis [26].

#### 2.3.4. Relative Age Effect Calculation

To characterize the sample’s chronological distribution, participants’ birth dates were collected and categorized into four birth quartiles (Q1, Q2, Q3, and Q4). According to the selection year, Q1 included players born in January, February, and March; Q2 included those born in April, May, and June; Q3 included those born in July, August, and September; and Q4 included those born in October, November, and December. The relative age effect (RAE) was calculated exclusively for descriptive purposes to provide a comprehensive overview of the players’ chronological profiles within the professional and non-professional environments, rather than to investigate its direct influence on physical performance outcomes.

#### 2.3.5. Statistical Analysis

Data were stratified by age category (U10–U14) and training environment (professional academy vs. non-professional club). Descriptive statistics (median, interquartile range (IQR)) were calculated for all anthropometric variables, including stature, body mass, body mass index (BMI), and physical performance measures (standing broad jump, T-test, and sit-and-reach).

To characterize the sample’s chronological distribution and identify potential selection biases, the presence of the Relative Age Effect (RAE) was examined using chi-square (χ^2^) goodness-of-fit tests, comparing the observed birth quartile distribution with an expected uniform distribution (25% per quartile) Effect sizes for χ^2^ were calculated using Cramér’s V. Quantile over(under)-representation was represented with Odds Ratios (OR) and their 95% Confidence Intervals.

Between-group differences in physical performance between professional and non-professional players were assessed within each age category using the Mann–Whitney U test. Effect sizes were calculated as r = Z/√N and interpreted as small (0.10), medium (0.30), and large (0.50).

The primary analysis focused on the association between biological maturation, expressed as years from aPHV, and physical performance outcomes. In particular, to examine whether these associations differed according to training environment and age category, a series of General Linear Models (GLMs) were performed investigating the presence of a three-way interaction between biological maturation, training environment and age category.

Given the non-normal distribution of several variables, as well as the presence of skewed distributions and heteroscedasticity across age categories, the original variables we applied Tukey’s Ladder of Powers to find the power transformation that best normalizes our outcome and independent variables; then the variables were standardized using z-scores.

More in detail, for each physical performance outcome, we fitted a model including training environment (professional vs. non-professional), age category (U10–U14), and standardized maturation status as predictors, together with all lower-order terms and the three-way interaction (Environment × Age Category × Maturation). To test the three-way interaction, we performed a Likelihood ratio test comparing models with and three way-interaction versus the model without interaction (e.g., containing only the main effects). The goodness of fit for each model was assessed using the Adjusted R-squared (R^2^). Model assumptions were rigorously assessed: normality of residuals was verified via Q-Q plots, while homoscedasticity was evaluated through Levene’s test and scatter plots of standardized residuals against predicted values. Influential cases were monitored using Cook’s Distance to ensure that no single observation disproportionately affected the models.

To facilitate interpretation of interaction effects, stratum-specific standardized coefficients were estimated from the fitted GLMs. These coefficients were obtained through linear combinations of the model parameters and represent the estimated association between maturation status and performance within each age category and training environment subgroup; these coefficients are reported on a standardized scale and can be interpreted as subgroup-specific measures of association.

As a sensitivity analysis, Spearman’s rank-order correlation coefficients (ρ) were also calculated separately within each subgroup defined by age category and training environment. These results are reported in the Supplementary Materials and were used to assess the consistency of the associations identified by the GLM approach. Association strength was interpreted as weak (|ρ| < 0.30), moderate (0.30 ≤ |ρ| < 0.50), and strong (|ρ| ≥ 0.50).

All statistical analyses were performed using IBM SPSS Statistics (version 29.0, IBM Corp., Armonk, NY, USA) and R (version 4.5.1) R Core Team (2025). _R: A Language and Environment for Statistical Computing_. R Foundation for Statistical Computing, Vienna, Austria. <https://www.R-project.org/>.

## 3. Results

### 3.1. Descriptive Overview

A total of 302 male youth football players (U10–U14) were included, comprising 122 players from a professional academy and 180 from non-professional clubs. Physical performance outcomes are summarized in Table 2.

### 3.2. Between-Group Differences in Physical Performance

**Figure 1:**
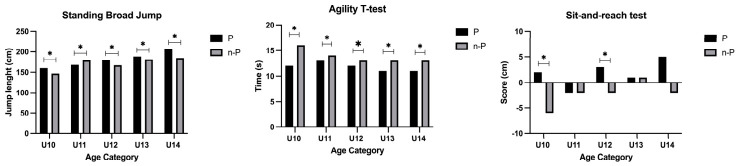
Comparison of physical performance metrics across age categories and training environments. The bar charts illustrate the mean scores for three functional tests stratified by age group (U10–U14) and training level (P: Professional academy; n-P: non-Professional academy). (**Left**) panel (Standing Broad Jump): Standing Broad Jump scores representing lower-limb explosive power in centimeters (cm). (**Middle**) panel (Agility T-test): Performance times for the agility T-test in seconds (s), where lower values indicate superior multidirectional speed. (**Right**) panel (Sit-and-reach test): Flexibility scores measured in centimeters (cm). Asterisk denotes a statistically significant difference between groups.

Between-group comparisons revealed significant differences in physical performance measures between players from professional and non-professional youth academies across multiple age categories. The data presented in Table 2 are plotted in Figure 1 to allow for a better visualization of the results.

In the Under-10 category, players from the professional youth academy demonstrated superior performance compared with their non-professional counterparts in standing broad jump (U = 737.5, *p* = 0.0019, r = 0.38), T-test agility (U = 1141, *p* < 0.001, r = 0.36), and sit-and-reach flexibility (U = 891.5, *p* < 0.001, r = 0.64), with medium to large effect sizes.

In the Under-11 category, significant differences were observed for standing broad jump (U = 285.5, *p* = 0.037, r = 0.27) and T-test performance (U = 1069, *p* < 0.001, r = 0.32), whereas no significant differences were detected for flexibility.

In the Under-12 category, players from the professional youth academy outperformed those from non-professional academies in standing broad jump (U = 949, *p* = 0.0085, r = 0.29), T-test performance (U = 155.5, *p* < 0.001, r = 0.60) and sit-and-reach performance (U = 1079.5, *p* < 0.001, r = 0.44).

In the Under-13 category, significant between-group differences were found for stature (U = 704, *p* < 0.001, r = 0.44), standing broad jump (U = 561, *p* = 0.040, r = 0.27), and T-test performance (U = 1324, *p* = 0.041, r = 0.19), all favoring the professional youth academy.

In the Under-14 category, players from the professional youth academy showed superior performance in standing broad jump (U = 226, *p* = 0.0048, r = 0.48), T-test agility (U = 47.5, *p* < 0.001, r = 0.77), and sit-and-reach flexibility (U = 239, *p* = 0.0011, r = 0.55), with medium to large effect sizes. These results highlight a consistent performance advantage associated with the training environment across all developmental stages.

### 3.3. Relationship Between Training Environment, Maturation, and Physical Performance

The GLM analysis revealed that the interaction between training environment, age category, and maturation status significantly predicted physical performance across all domains. The model for the T-test showed the highest predictive power, accounting for approximately 53% of the variance (Adjusted R^2^ = 0.53), with a highly significant three-way interaction (*p* < 0.001). In contrast, the model for the Standing Broad Jump (SBJ) explained 14% of the variance (R^2^ = 0.14), whereas yielding a significant three-way interaction (*p* < 0.001). Similarly, the model for the Sit and Reach explained a lower portion of the variance (R^2^ = 0.19), although the three-way interaction remained statistically significant (*p* = 0.013). Standardized β coefficients indicated that the association between maturation and performance was more pronounced in the non-professional setting compared to the professional academy, particularly for agility. These results confirm that the association between biological maturation and physical outcomes varies significantly depending on the training environment.

In the professional academy group, significant associations emerged only in the older age categories. Specifically, in U11 players, a moderate negative association was found between biological maturation and sit-and-reach performance (β = −0.477, *p* = 0.024). In U13 players, biological maturation was strongly and negatively associated with standing broad jump performance (β = −0.450, *p* = 0.039). No other significant associations were observed in the professional group across U10–U14 categories (*p* > 0.05).

In the non-professional academy group, significant associations were more frequent. In U11 players, biological maturation showed a moderate negative association with standing broad jump performance (β = −0.514, *p* = 0.001). In U12 players, a significant negative association was found with T-test performance (β = −0.255, *p* = 0.002). In U13 players, biological maturation was moderately and negatively associated with T-test performance (β = −0.305, *p* = 0.006). No significant associations were observed for sit-and-reach performance in the non-professional group (*p* > 0.05).

Taken together, these findings indicate that the relationship between biological maturation and physical performance is age- and context-dependent, with more consistent associations observed in non-professional youth players, particularly for agility-related performance.

### 3.4. Relative Age Distribution

**Figure 2:**
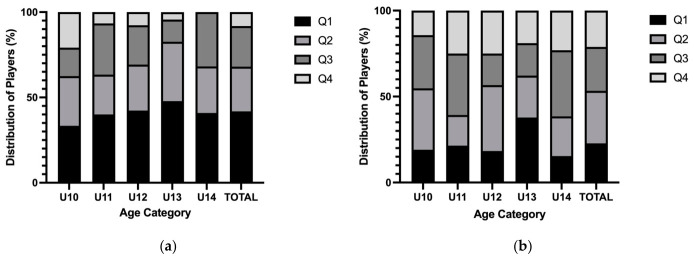
Graphical distribution of Relative Age Effect: (**a**) Relative distribution of birth quarters across age categories in professional youth soccer players. The stacked bar chart illustrates the percentage distribution of players according to their birth quarter (Q) within each age category (from U10 to U14) and for the total professional cohort. Birth quarters are defined as follows: Q1 (January–March), Q2 (April–June), Q3 (July–September), and Q4 (October–December). The Y-axis represents the relative frequency (%) of players within each subgroup. The visual highlights the presence of a Relative age effect (RAE), characterized by a higher representation of players born in the first half of the year (Q1 and Q2) compared to those born in the latter months (Q3 and Q4); (**b**) Relative distribution of birth quarters across age categories in non-professional youth soccer players. This figure depicts the frequency distribution (%) of birth quarters for players in the non-professional cohort across five age groups (U10–U14) and the total group. Each bar represents the cumulative 100% of the specific category, subdivided into Q1 (black), Q2 (dark grey), Q3 (medium grey), and Q4 (light grey). This figure serves to analyze the distribution of participation and potential selection biases in non-elite environments, providing a comparative baseline for the professional selection trends observed in the study.

The relative age distribution across age categories and training environments is illustrated in Figure 2. Chi-square goodness-of-fit analyses revealed a significant relative age effect in the professional youth academy in the U13 and U14 categories (*p* < 0.05), whereas no significant deviations from a uniform birth quartile distribution were observed in younger categories (U10–U12). In the non-professional youth academy, no significant relative age effect was detected in any age category.

## 4. Discussion

### 4.1. Training Environment as an Important Co-Determinant of Performance

The present findings indicate that physical performance differences between young football players were observed across training environments. However, because biological maturation, selection processes, and accumulated training exposure are likely interrelated, the independent contribution of the training environment cannot be determined within the present cross-sectional design. Across all age categories (U10–U14), players from the professional academy consistently outperformed their peers from non-professional clubs in explosive power (SBJ), agility (T-test), and flexibility (sit-and-reach), with small-to-large effect sizes depending on age category. These findings indicate that players competing within the professional academy demonstrated superior performance across several physical domains from early developmental stages onward [26,27,28].

Importantly, these performance differences were already evident in the youngest age groups (U10–U12), where players are predominantly pre-pubertal and maturational variability is expectedly limited compared to circa-pubertal stages [1]. This is also reflected in our sample data (Table 1), where the estimated age at peak height velocity (aPHV) for U10 to U12 cohorts shows a narrow distribution tightly bound within typical pre-pubertal ranges. One possible explanation is that differences in training exposure, coaching practices, competitive demands, or selection processes may contribute to these performance differences, although these factors were not directly measured [29,30,31]. Similar conclusions have been reported in youth football literature, where structured training environments have been shown to accelerate neuromuscular development independently of maturation-related growth processes [1].

A key observation from this analysis is the absence of significant correlations between aPHV and physical performance measures in the professional academy group, despite clear between-group performance differences.

The weaker maturation–performance associations observed within the professional academy may reflect several non-mutually exclusive explanations. First, professional academies are generally characterized by more structured developmental systems; however, specific training content and coaching practices were not quantified in the present study [32]. Resistance training, plyometric exercises, and movement skill development have been shown to enhance strength, power, and agility in pre- and circa-aPHV athletes [32,33].

Second, selection procedures within professional academies may differ from those used in non-professional environments, although the specific criteria adopted by the participating clubs were not formally evaluated. Previous research has shown that elite youth academies tend to retain later-maturing players when technical proficiency and game intelligence are prioritized, contributing to a more homogeneous physical profile [11,34].

In contrast, significant associations between aPHV and performance were observed in the non-professional clubs, particularly for explosive power (SBJ) and agility (T-test). These findings are consistent with previous literature showing that biological maturation is frequently associated with physical performance during youth development [1].

Differences in training characteristics between environments may contribute to these findings, although training quality and load were not assessed. As a result, early maturing players may disproportionately benefit, while later-maturing players may be temporarily disadvantaged despite similar technical potential [34,35].

This phenomenon has been widely described in youth sport and is closely related to maturational bias and relative age effects, whereby physically advanced players are more likely to be identified as talented due to superior short-term performance [34]. The present findings extend this concept by showing that the strength of the maturation–performance relationship differs between the evaluated environments. This difference is statistically supported by the highly significant three-way interaction (environment × category × maturation) found for the T-test (*p* < 0.001). By using standardized coefficients, we observed that the ‘maturity–performance’ association varies significantly across the two training contexts. These findings suggest that the relationship between maturation and performance may differ according to the developmental context in which players are embedded.

### 4.2. Integrating Category-Specific Results

The category-specific Mann–Whitney analyses further support this interpretation. From U10 to U14, professional group players showed consistently superior performance. While the magnitude of these differences fluctuated across intermediate developmental stages, the largest effect sizes for both explosive power and agility were observed in the oldest category (U14). Interestingly, these differences emerged despite the lack of a significant aPHV–performance relationship within the professional group, suggesting that factors beyond biological maturation may contribute to the observed performance differences [36].

In older categories (U13–U14), where maturational differences typically become more pronounced, the persistence of superior performance in professional players without corresponding aPHV correlations suggests that different developmental trajectories and selection histories may contribute to the weaker maturation–performance associations observed in the professional academy [37,38]. The T-test model explained a larger proportion of variance than the other performance outcomes. However, this finding should not be interpreted as evidence of a specific physiological mechanism, as agility performance is influenced by multiple factors including anthropometric characteristics, coordination, movement technique, neuromuscular function, and task familiarity. Conversely, the lower variance explained for both the SBJ (0.14) and the Sit and Reach (0.15) suggests that explosive power and flexibility are less dependent on the broad interaction of maturation and competitive level. Taken together, these findings indicate that physical performance differences emerge from a multifactorial interaction among maturation, anthropometric development, accumulated experience, training exposure, and contextual factors. The relative contribution of each component cannot be determined within the present study.

### 4.3. The Relative Age Effect

Relative age distribution in the present study was examined solely for descriptive purposes. A significant overrepresentation of early-born players was observed in the older categories of the professional academy, while no such pattern emerged in the non-professional setting.

Importantly, no differences in biological maturation or physical performance were observed across birth quartiles. No clear association between relative age distribution and performance outcomes emerged within the present sample; however, the study was not specifically powered to evaluate quartile-based performance differences.

These findings suggest that relative age should be interpreted as a contextual characteristic of the sample rather than a determinant of performance.

### 4.4. Practical and Theoretical Implications

From a practical perspective, these findings highlight the risk of overestimating the role of biological maturation in non-professional contexts, where performance may be disproportionately driven by PHV rather than trainable qualities. Coaches and practitioners working in such environments should interpret physical performance tests with caution and consider maturation status when evaluating youth players. The use of standardized coefficients, as suggested by our models, provides a quantifiable basis for ‘de-biasing’ talent identification. Given that the association of maturation with performance is not uniform but varies across environments and physical qualities, practitioners should transition toward maturation-adjusted performance metrics. This approach would ensure that selection is based on long-term technical potential rather than transient biological advantages, which our data shows to be particularly influential in less structured environments. Conversely, professional academy players demonstrated weaker maturation–performance associations than non-professional players; however, the mechanisms underlying these differences remain unclear. This supports long-term athlete development models that emphasize progressive training, movement competence, and technical mastery over early physical dominance [9]. It is also important to acknowledge that factors not assessed in the present study, including socioeconomic background, access to facilities, coaching expertise, family support, accumulated training history, and previous selection experiences, may contribute to differences observed between environments.

## 5. Conclusions

This cross-sectional study examined the relationships between biological maturation, physical performance, and training environment in youth football players. The findings indicate that associations between biological maturation and physical performance differed according to training environment. Stronger maturation–performance relationships were generally observed among players competing in non-professional settings, whereas weaker associations emerged within the professional academy environment.

These results suggest that the developmental context may influence how biological maturation is associated with physical performance during youth football development. However, because biological maturation, player selection processes, and accumulated training exposure are likely interrelated, the present study cannot determine the independent contribution of the training environment, nor can causal inferences be made regarding the mechanisms underlying these associations. The interpretation of these findings should also consider several limitations. Crucially, because specific variables such as training quality, coaching practices, accumulated training history, and socioeconomic factors were not directly measured, their independent contributions cannot be separated from the observed environmental differences. Additional limitations include the cross-sectional design, the absence of direct measures of body composition, and the constraints inherent to maturity estimation using predictive equations. Furthermore, the study focused exclusively on physical performance measures and did not assess technical, tactical, or psychosocial characteristics, which are also important determinants of long-term football development and success.

Taken together, these findings highlight the importance of considering environmental context when interpreting physical performance during youth development. Future longitudinal studies incorporating detailed measures of maturation, training exposure, selection history, and multidimensional performance outcomes are needed to clarify the complex interactions between biological development and the training environment in youth football.

## Figures and Tables

**Table 1:** Anthropometric characteristics by age category and training environment.

Age Category	Environment	Body Mass (kg)	Stature (cm)	BMI (kg·m^−2^)	aPHV (Years)
		Median ± IQR	Median ± IQR	Median ± IQR	Median ± IQR
U10	P (*n* = 24)	33.75 ± 6.90	140.00 ± 8.00	17.26 ± 1.96	13.33 ± 0.49
	n-P (*n* = 42)	31.75 ± 4.95	137.00 ± 6.00	17.10 ± 2.05	13.25 ± 0.57
U11	P (*n* = 30)	37.30 ± 6.30	145.00 ± 5.75	17.82 ± 2.08	13.64 ± 0.43
	n-P (*n* = 28)	39.50 ± 12.75	143.00 ± 7.75	19.28 ± 4.05	13.42 ± 0.56
U12	P (*n* = 23)	41.60 ± 11.35	151.00 ± 10.00	18.45 ± 3.09	13.91 ± 0.81
	n-P (*n* = 60)	40.20 ± 8.70	149.15 ± 13.00	18.03 ± 3.32	13.77 ± 0.74
U13	P (*n* = 23)	41.80 ± 12.35	158.00 ± 14.00	17.46 ± 2.08	14.07 ± 0.76
	n-P (*n* = 37)	45.00 ± 14.00	150.00 ± 16.00	18.60 ± 7.24	14.59 ± 0.84
U14	P (*n* = 22)	58.50 ± 11.25	171.50 ± 8.25	19.64 ± 1.10	13.73 ± 1.09
	n-P (*n* = 13)	43.00 ± 8.00	158.00 ± 8.50	17.80 ± 2.29	14.90 ± 0.56

**Table 2:** Physical performance measures by age category and training environment.

Age Category	Environment	Standing Broad Jump (cm)	T-Test (s)	Sit-and-Reach Test (cm)
		Median ± IQR	Median ± IQR	Median ± IQR
U10	P	159.50 ± 16.75	11.52 ± 0.97	2.00 ± 4.25
	n-P	148.00 ± 22.25	15.47 ± 2.05	−5.50 ± 8.00
U11	P	167.50 ± 11.50	12.75 ± 0.66	−1.00 ± 7.50
	n-P	179.50 ± 22.25	13.84 ± 1.28	0.00 ± 8.25
U12	P	180.00 ± 15.00	11.75 ± 0.57	3.04 ± 4.00
	n-P	170.00 ± 24.00	12.90 ± 1.52	0.00 ± 7.25
U13	P	186.00 ± 14.00	10.82 ± 0.47	2.00 ± 8.50
	n-P	181.00 ± 21.00	13.48 ± 2.00	2.00 ± 8.00
U14	P	208.50 ± 25.25	10.85 ± 0.70	7.00 ± 7.75
	n-P	174.92 ± 34.00	13.00 ± 1.52	0.00 ± 6.00

## Data Availability

The data presented in this study are available on request from the corresponding author.

## Supplementary Materials

The following supporting information can be downloaded at: https://www.mdpi.com/article/10.3390/jfmk11030257/s1, Table S1: GLMs results.
