# Supplementary material for: Biological Maturation and Physical Performance in Youth Football: Associations Across Professional and Non-Professional Environments
Source: J Funct Morphol Kinesiol. 2026 Jun 29;11(3):257. doi: 10.3390/jfmk11030257 (PMC13397898; doi:10.3390/jfmk11030257)
Supplement: Supplementary file 1 [file jfmk-11-00257-s001.zip › jfmk-4320504-supplementary.pdf]

Standardized coefficients, p values								PHV		
	professional (P)			non-professional (n-P)				Tukey's Ladder of Powers	lambda	-3,775
**U10**	Standardised coefficient	95%CI	p value	Standardised coefficient	95%CI	p value		Shapiro test	p value	0,638
SBJ distances (cm)	-0,018	(-0,399; 0,377)	0,925	0,134	(-0,146; 0,413)	0,347				
T-Test (s)	0,032	(-0,236; 0,299)	0,816	-0,078	(-0274; 0,118)	0,435		Modello SBJ		
Sit-and-Reach Test	0,040	((-0,334; 0,415)	0,831	0,177	(-0,098; 0,452)	0,205		Tukey's Ladder of Powers	lambda	0,525
**U11**								Shapiro test	p value	0,291
SBJ distances (cm)	-0,091	(-0,510; 0,328)	0,670	-0,514	(-0,809; -0,212)	0,001		Test for interaction	p value	<0,0001
T-Test (s)	-0,117	(-0,412; 0,117)	0,433	0,112	(-0,095; 0,319)	0,286		Adjusted R squared		0,137
Sit-and-Reach Test	-0,477	(-0,889; -0,065)	0,024	-0,211	(-0,501; 0,078)	0,152				
**U12**								Modello T-Test		
SBJ distances (cm)	-0,225	(-0,623; 0,174)	0,268	-0,082	(-0,312; 0,149)	0,486		Tukey's Ladder of Powers	lambda	-1,375
T-Test (s)	-0,130	(-0,403; 0,150)	0,363	-0,255	(-0,417; -0,093)	0,002		Shapiro test	p value	0,125
Sit-and-Reach Test	-0,177	(-0,569; 0,215)	0,375	-0,099	(-0,326; 0,128)	0,392		Test for interaction	p value	0,006
**U13**								Adjusted R squared		0,564
SBJ distances (cm)	-0,450	(-0,450; -0,021)	0,039	0,165	(-0,144; 0,475)	0,294				
T-Test (s)	-0,013	(-0,314, 0,288)	0,931	-0,305	(-0,522; -0,088)	0,006		Modello Sit-and-Reach Test		
Sit-and-Reach Test	-0,444	(-0,866; -0,023)	0,049	-0,203	(-0,507; 0,101)	0,190		Tukey's Ladder of Powers	lambda	1,325
**U14**								Shapiro test	p value	0,002
SBJ distances (cm)	-0,481	(-0,890; 0,107)	0,126	0,628	(-0,036; 1,290)	0,064		Test for interaction	p value	<0,0001
T-Test (s)	-0,086	(-0,430; 0,257)	0,620	-0,463	(-0,929; 0,002)	0,051		Adjusted R squared		0,169
Sit-and-Reach Test	0,099	(-0,382; 0,579)	0,686	0,318	(-0,334; 970)	0,338				

Coefficienti di correlazione, p values				
	professional (P)		non-professional (n-P)	
**U10**	Correlation coefficient	p value	Correlation coefficient	p value
SBJ distances (cm)	0,045	0,835	0,072	0,652
T-Test (s)	0,036	0,867	-0,076	0,631
Sit-and-Reach Test	0,171	0,424	0,224	0,154
**U11**				
SBJ distances (cm)	-0,106	0,576	-0,459	0,014
T-Test (s)	-0,104	0,583	0,143	0,469
Sit-and-Reach Test	-0,454	0,012	-0,282	0,146
**U12**				
SBJ distances (cm)	-0,300	0,164	-0,086	0,514
T-Test (s)	-0,129	0,556	-0,304	0,018
Sit-and-Reach Test	-0,212	0,331	-0,078	0,555
**U13**				
SBJ distances (cm)	-0,658	0,001	0,208	0,217
T-Test (s)	0,111	0,615	-0,453	0,005
Sit-and-Reach Test	-0,344	0,108	-0,117	0,489
**U14**				
SBJ distances (cm)	-0,370	0,090	0,500	0,085
T-Test (s)	-0,171	0,447	-0,633	0,020
Sit-and-Reach Test	0,111	0,623	0,120	0,697
